# Efficacy of single-layer continuous suture of the posterior wall in anastomosis involving a difficult location of the digestive tract

**DOI:** 10.3892/ol.2014.2397

**Published:** 2014-07-30

**Authors:** GUO-CAI LI, YONG XU, YU-CHUN ZHANG, FANG-CHENG ZHANG, QI WANG, QING-JIU MA

**Affiliations:** Department of General Surgery, Xi’an Gao Xin Hospital, Xi’an Jiao Tong University, Xi’an, Shaanxi 710075, P.R. China

**Keywords:** anastomosis, suture, single-layer, digestive tract, complication

## Abstract

Surgery for digestive tract disease predominantly consists of reconstruction and anastomosis. Due to the difficult location, anastomosis is extremely challenging and the risk of complication increases accordingly. Traditional manual anastomosis and the application of a stapling device are insufficient. Therefore, the aim of this study was to investigate the feasibility and safety of a novel manual method in a difficult anastomotic location, consisting of a single-layer continuous suture in the posterior wall. In total, 15 beagle dogs were included in the study; eight underwent surgery with the novel manual method for reconstruction and anastomosis of the digestive tract, while seven underwent surgery with the stapler device as a control. The subsequent postoperative complications were observed and, three months later, the anastomotic ports were excised, and the pathological formation and morphological changes were evaluated. No statistically significant differences were identified between the total (50.0 vs. 57.1%; P=0.782) and anastomotic (0.0 vs. 28.6%; P=0.200) complication rates in the manual suture and staple suture groups, respectively. Compared with the control group, the operative expenditure was lower in the manual group (1726.7±33.5 vs. 2135.7±43.1 renminbi; P=0.001), the diameter of the anastomotic port was larger in the manual group (3.04±0.07 vs. 2.24±0.25 cm; P=0.004) and the thickness of the anastomotic port (in cm) was thinner in the manual group (2.94±0.06 vs. 5.07±0.85; P=0.002). Furthermore, the pathological formation of the anastomositic port in the manual group was improved. The results of the current study suggest single-layer continuous suture of the posterior wall in anastomosis of the digestive tract to be a novel method with feasibility and safety, particularly in difficult anastomotic locations.

## Introduction

Surgery for digestive tract disease predominantly consists of reconstruction and anastomosis (1). The methods of anastomosis influence the outcome of surgery, postoperative quality of life and complications.

Traditional manual anastomosis is a complex surgery, consisting of double-layer interrupted suture, which requires an experienced surgeon, and has a higher incidence of postoperative complication. The incidence of stricture and leakage in traditional colorectal anastomosis is 4.5 and 1.8%, respectively (2). Furthermore, in certain types of surgery, such as esophagogastrostomy and colorectostomy, the surgery is more difficult due to special anatomic location and poor exposure of the back wall of the anastomosis, increasing the risk of complication accordingly.

In 1909, the first surgical stapler was developed and primarily used for dividing and stapling bowel segments (3). With the general application of the stapling device, an increasing number of proximal gastric resections via the abdomen have been performed for cardiac cancer. The stapling device has contributed to reduced surgery times in difficult locations (4) and decreased the rates of edema (5), infection (6), leakage and stricture of anastomosis (7), as well as morbidity of pulmonary complication (8). However, limitations of this method exist, and improper manipulation may cause partial incisions and tearing, which result in leakage and bleeding of the anastomosis (9,10). In addition, irregular suture of the mucosa may cause hyperplasia of granulation tissue and scar formation, which further increase the risk of stricture (11,12). In certain cases, with preoperative obstruction of the digestive tract, mucosal edema, thickened muscle layer and dysfunctional healing, the application of stapling device is constricted. The stapling techniques have been criticized in view of their apparent expense. Therefore, the identification of a novel manual method with simple and convenient characteristics for reconstruction in difficult locations is beneficial.

Single-layer continuous suture is a common method of blood vessel surgery (13). The advantage of this approach is its simplicity, and is particularly suited for vessel anastomosis in deep tissues. Single-layer suture has been confirmed to be tight and safe, similar to double-layer suturing in gastrointestinal anastomosis (14,15), and superior to conventional suturing in the colon (4). However, the efficacy of the method combined with continuous suture in difficult anastomotic locations remains unknown.

In the present study, prospective and controlled experiments were performed, including single-layer continuous suture of the posterior wall, and double-layer interrupted suture of the anterior wall. The aim was to investigate the efficacy of the novel manual method in difficult anastomotic locations, and the method was found to be feasible and safe. This novel method may simplify the approach in complex surgery as a result of the special anatomic sites, and reduce expenditure.

## Materials and methods

### Animals

In total, 15 beagle dogs, including 10 females and five males, with a median weight of 9 kg (range, 7–12 kg), were included in the study. The dogs were fed with specialized dog food and supplied with an appropriate amount water. The dogs were divided into two groups: A manual group and a staple group. The manual group underwent single-layer continuous suture, while the staple group underwent suture with a stapler device. The study was approved by the Xi’an Medical Experimental Animal Care Commission (Xi’an, China).

### Procedures

The dogs were anesthetized by an intravenous injection of pentobarbital sodium (30 mg/kg; Sigma-Aldrich China, Inc., Shanghai, China), and an intratracheal tube was inserted and connected to a respirator during the surgery. A total of 800,000 IU of penicillin and 250 mg of metronidazole (both North China Pharmaceutical Group Corporation, Shanghai, China) were administered intravenously at the initiation and end of the anesthesia.

### Esophagogastric anastomosis

All animals were fasted for one day prior to the surgery. Following disinfection, a laparotomy was performed through an upper midline abdominal incision. The distal esophagus and gastric fundus were subsequently disassociated and removed. The approach of the staple suture was similar to that performed on human patients. Briefly, a purse-string suture was inserted and the anvil of a circular staple was introduced into the distal esophageal end. Next, the central shaft of the stapler (Q/CYAE561-2001, Shanghai Medical Instruments (Group) Ltd., Shanghai, China) was inserted through the anterior wall of the gastric fundus and the anvil (outer ring) was refitted onto the shaft. An end-to-side esophagogastric anastomosis was created with the button, the stapler was withdrawn and the residual end of the gastric fundus was closed with sutures. For the manual suture, the lesser curvature of the proximal stomach was closed with silk stitch, and the greater curvature was prepared for end-to-end anastomosis. The posterior wall of the anastomosis was then closed by single-layer continuous suture using 4-0 Prolene threads (Ethicon, Inc., Somerville, NJ, USA), and the priority wall was conventionally sutured by double-layer interrupted suture using silk thread (total layer suture combined with embedding of the serosa and muscle tissue).

### Colon-to-rectum anastomosis

Bowel preparation consisted of fluids by mouth for three days prior to surgery. Laparotomy was performed through a midline incision, and the distal colon and rectum were disassociated. A circumferential dissection of the rectum was undertaken to the level of 6 cm from the dentate line, and ~5 cm of the rectum was resected. For the manual suture, the posterior wall of the anastomosis was sutured by single-layer continuous suture with 4-0 Prolene thread, and the priority wall was sutured by double-layer interrupted suture with silk thread as usual. For the staple suture, a purse-string suture was inserted and the anvil was placed on the proximal end of the rectum, while the distal colon end was used to introduce the staple. The central shaft of the stapler was then inserted through the antimesenteric border of the colon and the anvil was refitted. An end-to-side esophagogastric anastomosis was created with the button, the stapler was withdrawn and the distal colon end was closed with suture, while the abdominal wound was sutured in layers.

Following surgery, the dogs received intravenous injections of electrolyte solution and antibiotics for two days. On the third postoperative day, the dogs were allowed to drink water and a fluid meal.

### Observations

Prior to and for 0.5 months following surgery, the anal temperature of each dog was measured every two days, and prior to and for three months following surgery, the feeding amount and weights were measured once a week. The complications of surgery, including wound infection, stricture and leakage of the anastomosis, were observed according to symptoms and signs.

### Blood samples

Prior to and one month following surgery, blood samples were obtained from a rear-leg vein and drawn into ice-chilled glass tubes containing EDTA-2Na (1.25 mg/ml blood). Regular blood and liver function tests were then performed under the appropriate circumstances.

### Specimen collection

At three months following surgery, each dog was anesthetized with pentobarbital sodium and laparotomized on the day following a 24-h fast. Next, full-thickness tissue specimens were obtained from the anastomotic port. The diameter of the anastomotic port and thickness of the wall were measured. In total, four tissue specimens were dissected from each anastomotic port symmetrically, and then dipped into 10% formalin solution. The tissues were embedded in paraffin, sectioned (3 μm thick) along the longitudinal axis of the intestine and stained with hematoxylin and eosin (H&E).

### Morphological examinations of the anastomotic port

All slices with H&E staining were evaluated for the severity of inflammation, stricture and fibrosis by two independent pathologists under a light microscope (x100 magnification; Q550cw, Leica, Mannheim, Germany) of 10 fields. The standard scores were determined as follows: i) inflammation: 1, small amount of lymphocytes or granulocytes observed in <4 fields; 2, medium amount of lymphocytes or granulocytes observed in 4–7 fields; and 3, large amount of lymphocytes or granulocytes observed in >7 fields; ii) fibrosis: 1, small amount of fibrocytes; 2, medium amount of fibrocytes; and 3, large amount of fibrocytes; iv) irregular structure: 1, slight; 2, moderate; and 3, severe.

### Statistical methods

Data were analyzed using SPSS 11.5 software (SPSS Inc., Chicago, IL, USA). Student’s t-test was used to analyze the differences between weight, temperature, feeding amount, surgery time and amount of bleeding. Fisher’s exact test was used for the comparison between the incidence rate and complications. All statistical tests were two-sided and P<0.05 was considered to indicate a statistically significant difference.

## Results

### Total status of animal

A total of 15 dogs underwent surgery successfully. In the manual group, eight dogs underwent single-layer continuous suture of the posterior wall in esophagogastric anastomosis or colon rectum anastomosis, while in the staple group, seven dogs underwent staple anastomosis as controls. Following three months of observation, intra- and postoperative complications were identified, including bleeding, shock, leakage or stricture of anastomosis and infection (Table I).

### Weight, temperature and diet

**Figure 1:**
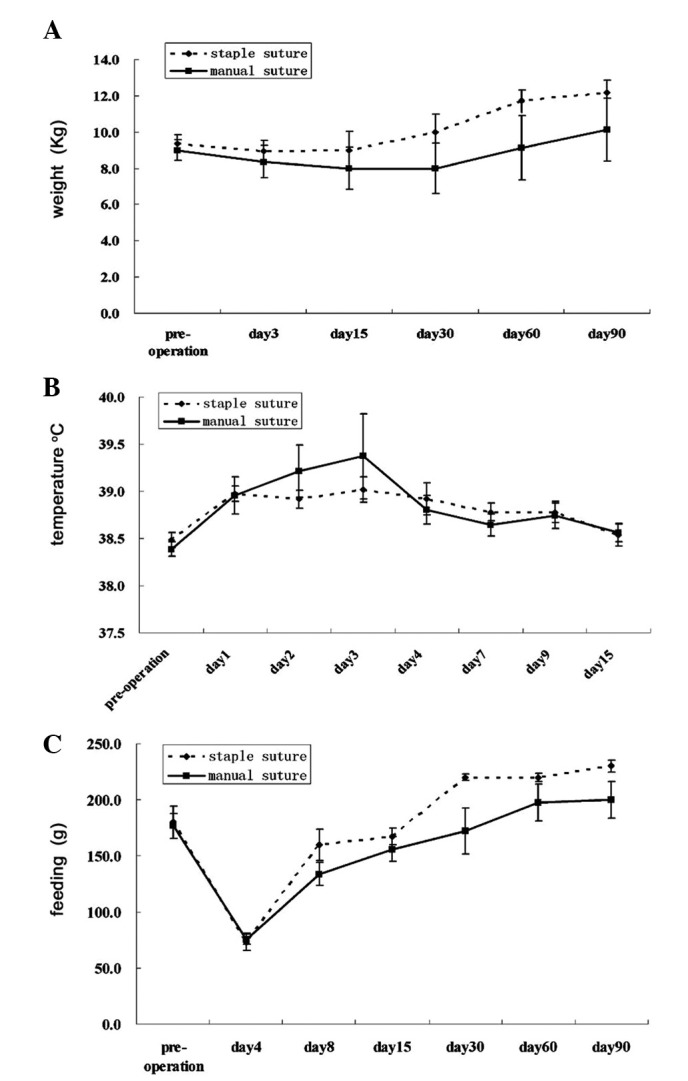
Observations with regard to animal weight, temperature and feeding pre- and post-surgery. (A) The growth curves of the animals’ weight; no significant difference was identified between weight in the manual and staple groups. (B) The growth curves of the animals’ temperature; no significant difference was identified between temperature in the manual and staple groups. (C) The growth curves of animals’ feeding; no significant difference was identified between feeding in the manual and staple groups.

Prior to surgery, the mean weight of animals in the manual group was 9.0±0.6 kg, while this was 9.4±0.5 kg (P=0.658) in the staple group. At three months following surgery, the mean weight of the animals in the manual and suture groups was 10.1±1.7 and 12.2±0.7 kg, respectively (P=0.367; Table II). Similarly, no significant difference was identified between the mean weights of the two groups at other observation time points (Fig. 1A). Prior to surgery, the mean temperature of the animals in the manual group was 38.4±0.1°C, while this was 38.5±0.1°C (P=0.392) in the staple group. At three days following surgery, the mean temperature of the manual and suture groups was 38.8±0.2 and 38.9±0.2, respectively (P=0.628). No significant difference was identified between the mean temperature in the two groups at other observation time points (Fig. 1B). Prior to surgery, the mean feeding amount of animals in the manual group was 176.7±10.9 g, and 180±14.1 g in the staple group (P=0.852). At one month following surgery, the mean feeding amount of the animals in the two groups was 172.0±20.3 versus 220±3.0 g (P=0.127). No significant difference was identified between the feeding amount of the two groups at other observation time points. (Fig. 1C).

### Surgery time, amount of bleeding and expenditure

The mean surgical time in the manual group was 2.0±0.1 h, while this was 1.9±0.2 h in the staple group (P=0.915). The mean volume of blood lost during surgery was 132.2±23.9 ml in the manual group and 151.4±36.7 ml in the staple group (P=0.655) (Table II). The surgical expenditure included the apparatus, stapler, thread and drugs. The total surgical expenditure in the manual group was significantly lower than that of the control group; 1726.7±33.5 versus 2135.7±43.1 renminbi (P=0.001) (Table II).

### Blood test

Blood tests were performed prior to and one month following surgery. In the manual and staple groups, no significant difference was identified between the routine blood and liver function tests (Table III).

### Complications of anastomosis

**Figure 2:**
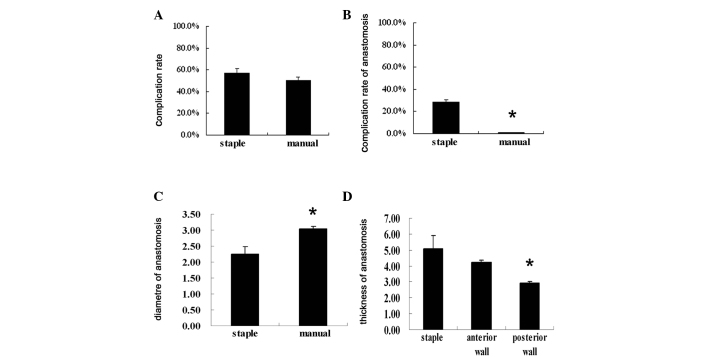
Evaluation between complications and anastomotic ports. (A) Compared with the staple group (controls), the complication rate in the manual group was reduced, but the difference was not significant. (B) The complication rate of anastomosis in the manual group was significantly lower than that in the controls. ^*^P<0.05, vs. the staple group. (C) The diameter of the anastomotic port in the manual group was larger than that in the controls. ^*^P<0.05, vs. the staple group. (D) The thickness of the posterior wall in the manual group was smaller than that in the anterior wall of the manual group and the walls of the controls. ^*^P<0.05 vs. the anterior wall of the manual group and the staple group. Data are presented as the mean ± standard error of the mean.

The incidence rate of the total complications in the manual group was 50%, and 57.1% in the staple group (P=0.782). The complications involving the anastomosis port were 0 and 28.6%, with no significant difference (P=0.200) (Fig. 2A and B and Table IV).

### Morphological changes in the anastomotic port

**Figure 3:**
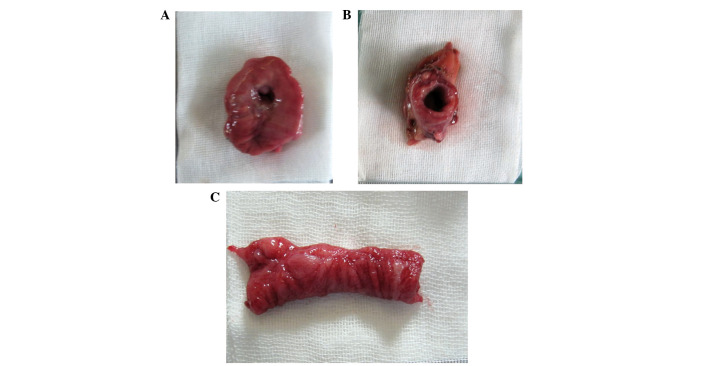
Tissues of the anastomotic port. (A) The anastomotic port of animal 3 following esophagogastric anastomosis with staple suture. The image shows stricture of the anastomotic port, with increased thickness and a diameter of 0.8 cm. (B) The anastomotic port of animal 15, following esophagogastric anastomosis with manual suture. The image shows a larger diameter and increased thickness of the anastomotic port. (C) The transversal inner surface of the anastomotic port following single-layer continuous suture.

The diameter of the anastomotic port in the manual group was 3.04±0.07 cm, while this was 2.24±0.25 in the staple group (P=0.004). The thickness of the anastomotic port in the posterior wall of the manual group was 2.94±0.06 cm, which was thinner than that of the staple group (5.07±0.85 cm) (P=0.002), and also thinner than in its anterior wall (4.22±0.16 cm) (P=0.036). However, no significant difference was identified between the staple group and the anterior wall of the manual group (P=0.179; Figs. 2CD and 3A–C). Anastomotic stricture was also identified in the staple group, with a diameter of <1 cm, while the thickness of the wall was 1 cm (Fig. 3A).

### Pathological formation of the anastomotic port

**Figure 4:**
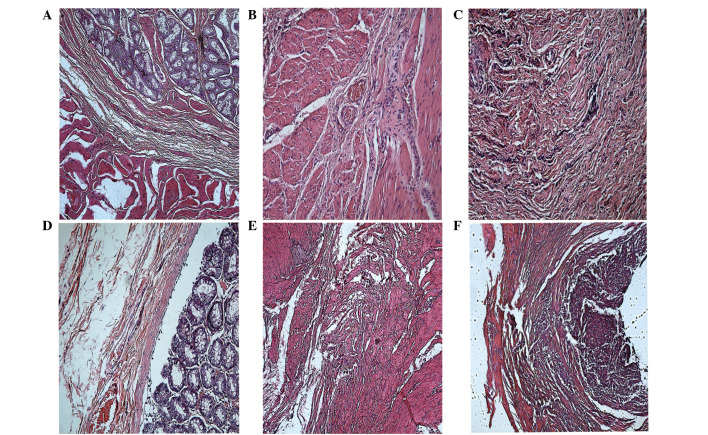
Hematoxylin and eosin staining of the tissues from the anastomotic port (magnification, ×20). (A) Normal esophageal tissue. (B) The stricture of the tissues from the posterior wall of the manual staple group following gastroesophageal anastomosis was irregular, and a section of fibrosis was found to embed the muscle layer, but no inflammation were observed. The slice was selected from sample 13. (C) Compared with the manual group, the stricture of the tissues in the staple group was irregular with fibrosis, and amounts of fibrocystic cells were found. (D) Normal tissue of the rectum. (E) The stricture of the posterior wall tissues of the manual staple group following colon-rectum anastomosis was irregular, with no evidence of fibrosis or inflammation. The slice was selected from sample 8. (F) A large amount of lymphocytes and granulated cells had infiltrated into the four-layer tissue of the anastomotic port in the staple group. The slice was selected from sample 5.

Compared with the staple group, inflammation of the anastomotic port was marginal in the manual group, with scores of 2.10±0.97 versus 1.58±0.83 (P=0.063), and the stricture was neater than that in the controls (2.40±0.75 vs. 1.79±0.88; P=0.020). The total score of the posterior wall was lower than that of the anterior walls, which were sutured by double-layer interrupted suture with silk thread (9.50±2.51 vs. 11.00±2.00; P=0.030) (Fig. 4A–F and Table V).

## Discussion

In this study, single-layer suture was combined with continuous suture in back wall anastomosis at challenging surgical sites. The study showed that this combined suture is technically possible to perform, and that, under experimental conditions, the novel anastomosis appears to be as safe as stapled sutures. The postoperative complications were reduced or the same as those in stapled sutures; however, the expenditure was evidently reduced.

The predominant complications of anastomosis are leakage, stricture and infection. To date, the superiority of staple suture has remained controversial. It has been reported that in previous hand-sewn and staple groups, the incidence of leakage following esophagogastric anastomosis is 0–21.9 and 0–25.8%, respectively, and 0–19.5% and 0–32.8%, respectively, for stricture (16–24). In rectal cancer, leakage following low anterior resection with the double stapling technique is 2.6–17% (25–28), and no significant difference has been identified between the morbidity or mortality rates in hand-sewn and stapled techniques (14,29). The results of the current study demonstrated that the incidence rate of total complications was 50%, and anastomosis complication was 13.3%. The results were higher than those identified in the previously described studies, but lower than a study which reported postoperative minor complications in 70.9% of patients and serious complications in 22.6% of patients following esophagogastric anastomosis (30). The reasons of complication in the present study may have also involved the following: i) Preoperative preparation, due to the challenges of bowel preparation and implantation of the gastric pipe; and ii) insufficient nutrition supply for animals following surgery. However, in the same conditions, the manual group demonstrated a lower rate of complications and higher safety than the staple group, providing evidence for its advantage.

The predominant challenges of anastomosis are due to exposure, particularly for anastomosis of the back wall in difficult locations. The anastomosis becomes invisible due to blockade by surrounding tissues, and even in the course of staple suture, the proceeding is not visible. However, continuous suture does not require a butt joint, which increases exposure to the back wall and contributes to convenience and safety.

Mechanical integrity and tissue viability are emphasized in gastrointestinal anastomosis. It has been shown in an experimental and comparative study, that single-layer anastomosis is as strong as two-layer suturing in the small intestine and colon, and may guarantee mechanical integrity (19). Tissue viability has also been found to closely correlate with a healthy blood supply and good nutritional status of suture line (7,10). The blood flow is always reduced in the suture line compared with the normal mucosa, and of the three anastomotic methods (stapled suture, two-layered manual and single-layered manual suture), the blood flow of suture line increases in turn (9,10) and, therefore, the mechanism requires further investigation.

In the present study, no significant difference was identified between surgical time in the manual and staple suture groups. Continuous suture contributes to reduced surgical time, and does not increase complication in episiotomy (31). Staple anastomosis reduces surgical time by ≤30 min due to cutting and suturing tissues only once (32); however, to acquire a safe anastomosis, a sharp blade and a sufficient amount of tissue cutting is required, which may lead to surgical issues and prolong surgery time.

The complication of stricture has been found to closely correlate with the diameter of the anastomosis and the thickness of the wall. Few studies regarding anastomotic ports have included morphological observations. In the current study, a smaller diameter and increased thickness of the anastomotic port were identified in the staple group, with the thickness of the anastomotic wall in one sample reaching 1 cm, and a narrow diameter of <0.8 cm. Staple suture is a double-layer suture and, in certain cases, an additional strengthening suture is required to reinforce the suture with a third layer; however, this may cause too much tissue to turn inwards and induce stricture formation (10). Staplers with several external diameters allow the resection of various diameters and the dissection of various surface areas (33). However, selecting a stapler is almost impossible due to fixed types. By contrast, single-layer suture causes little inward tissue movement, which may reduce the anastomotic stricture, and continuous suture has been confirmed to contribute to the adjustment of the anastomotic diameter (34).

Fibrosis is an important factor for anastomosis stricture, and, in this study, a decreasing number of inflammation and fibroblast cells were identified by microscope, as well as a slightly irregular structure in the novel manual suture group compared with the staple suture and conventional manual suture groups. These results provided evidence that manual sutures may efficiently reduce anastomotic complication, and support the view that staple sutures increase the rate of postoperative anastomotic stricture (21). The results are consistent with a meta-analysis of randomized and controlled trials, comparing hand-sewn with stapled esophagogastric anastomosis in other studies (7).

In conclusion, the results of the current study suggest that single-layer continuous suture in anastomosis of the posterior wall of the digestive tract is a novel method with feasibility and safety. This novel method simplifies the surgical approach and can be easily applied clinically, in particular, it can be used in challenging anastomosis of special anatomic sites. In addition, this method reduces expenditure and deserves generalization in the future.

## Figures and Tables

**Table I:** Surgical methods and survival of total animals.

n	Group	Anastomosis	Complication (yes/no)	Postoperative complication
1	Staple	EG	Yes	Infection and jaundice
2	Staple	EG	Yes	Bleeding and shock
3	Staple	EG	Yes	Stricture of anastomosis
4	Staple	EG	Yes	Leakage and infection
5	Staple	CR	No	
6	Staple	CR	No	
7	Staple	CR	No	
8	Manual	CR	No	
9	Manual	CR	No	
10	Manual	CR	Yes	Infection
11	Manual	EG	Yes	Bleeding and shock
12	Manual	EG	Yes	Infection
13	Manual	EG	No	
14	Manual	EG	No	
15	Manual	EG	Yes	Infection

EG, esophagogastric anastomosis; CR, colon-to-rectum anastomosis.

**Table II:** Preoperative, operative and postoperative observations.

Variables	Manual suture (mean ± SD)	Staple suture (mean ± SD)	P-value
n	8	7	
Weight, g			
Preoperative	9.0±0.6	9.4±0.5	0.658
Postoperative (3 months)	10.1±1.7	12.2±0.7	0.367
Temperature			
Preoperative	38.4±0.1	38.5±0.1	0.392
Postoperative (third day)	38.8±0.2	38.9±0.2	0.628
Feeding, g			
Preoperative	176.7±10.9	180±14.1	0.852
Postoperative (1 month)	172.0±20.3	220±3.0	0.127
Bleeding, ml	132.2±23.9	151.4±36.7	0.655
Surgery time, h	2.0±0.1	1.9±0.2	0.915
Expenditure (RMB)	1726.7±33.5	2135.7±43.1	0.001

Student’s t-test was used to assess the statistically significant difference between tumor volume in the manual suture and staple suture groups. SD, standard deviation; RMB, renminbi.

**Table III:** Comparison between laboratory tests in the staple and manual groups.

	Preoperative (mean ± SD)			Postoperative (mean ± SD)		
		
Variables	Staple	Manual	P-value	Staple	Manual	P-value
Blood routine examination						
WBC	6.99±2.40	6.44±2.99	0.70	7.07±2.11	7.44±4.09	0.89
HCT	40.77±7.80	39.59±7.44	0.76	38.07±7.70	37.68±9.55	0.95
RBC	5.94±1.10	5.69±1.01	0.64	6.29±1.37	6.59±0.95	0.72
Hb	143.00±26.94	136.67±24.49	0.63	133.67±23.86	148.40±21.31	0.40
PLT	160.00±139.05	245.11±130.54	0.23	226.00±87.07	446.0±249.51	0.20
Liver function						
T.BIL	2.42±0.50	2.09±0.74	0.40	2.33±0.59	2.14±0.74	0.71
ALT	48.40±15.60	40.13±7.22	0.22	45.67±9.87	52.00±12.63	0.49
AST	56.60±14.66	52.25±20.94	0.69	59.33±26.08	56.60±15.66	0.86
TBA	60.68±4.62	59.65±7.40	0.79	65.47±3.18	60.92±8.86	0.44
ALB	29.76±2.82	28.88±3.36	0.64	22.10±5.63	23.26±2.72	0.70
GLB	30.92±3.73	30.78±5.36	0.96	43.37±7.14	37.66±6.82	0.30
A/G	0.98±0.15	0.95±0.14	0.72	0.53±0.21	0.64±0.09	0.34

Student’s t-test was used to assess the statistically significant difference between tumor volume in the manual suture and staple suture groups. SD, standard deviation; RBC, red blood cell count; WBC, white blood cell count; HCT, hematocrit; Hb, hemoglobin; PLT, platelet count; T.BIL, total serum bilirubin; ALT, glutamic pyruvic transaminase; AST, glutamic oxaloacetic transaminase; TBA, serum protein; ALB, albumin; GLB, globulin; A/G, ratio of albumin to globulin.

**Table IV:** Complication of the two groups.

Variables	Manual, n (%)	Staple, n (%)	P-value
n	8 (100.0)	7 (100.0)	
Bleeding shock	1 (12.5)	1 (14.3)	0.919
Leakage	0 (0.0)	1 (14.3)	0.467
Stricture	0 (0.0)	1 (14.3)	0.467
Infection of abdominal	2 (25.0)	2 (28.6)	0.662
Infection of wound	1 (12.5)	1 (14.3)	0.919

Fisher’s exact test was used to assess the statistical difference between the manual and staple suture groups.

**Table V:** Pathological formation of the anastomotic port between the two groups.

Group	Score (mean ± SD)	P-value
Fibrosis		
Manual	1.92±0.78	0.354
Staple	2.15±0.88	
Inflammation		
Manual	1.58±0.83	0.063
Staple	2.10±0.97	
Stricture		
Manual	1.79±0.88	0.020
Staple	2.40±0.75	
Total of slice		
Manual	5.29±0.70	0.018
Staple	6.65±1.95	
Total of samples		
Manual	21.17±4.75	0.171
Staple	26.60±7.33	
Anterior-posterior		
Anterior wall of manual	11.00±2.00	0.030
Posterior	9.50±2.51	

Student’s t-test was used to assess the statistical significance between tumor volume in the manual and staple suture groups. SD, standard deviation.
